# HIV-1 resistance conferred by siRNA cosuppression of CXCR4 and CCR5 coreceptors by a bispecific lentiviral vector

**DOI:** 10.1186/1742-6405-2-1

**Published:** 2005-01-13

**Authors:** Joseph Anderson, Ramesh Akkina

**Affiliations:** 1 Dept Microbiology, Immunology and Pathology, Colorado State University, Fort Collins, Colorado 80523, USA

**Keywords:** HIV/AIDS gene therapy, HIV-1 co-receptors, CCR5 siRNA, CXCR4 siRNA, Bispecific Lentiviral vector

## Abstract

**Background:**

RNA interference (RNAi) mediated by small interfering RNAs (siRNAs) has proved to be a highly effective gene silencing mechanism with great potential for HIV/AIDS gene therapy. Previous work with siRNAs against cellular coreceptors CXCR4 and CCR5 had shown that down regulation of these surface molecules could prevent HIV-1 entry and confer viral resistance. Since monospecific siRNAs targeting individual coreceptors are inadequate in protecting against both T cell tropic (X4) and monocyte tropic (R5) viral strains simultaneously, bispecific constructs with dual specificity are required. For effective long range therapy, the bispecific constructs need to be stably transduced into HIV-1 target cells via integrating viral vectors.

**Results:**

To achieve this goal, lentiviral vectors incorporating both CXCR4 and CCR5 siRNAs of short hairpin design were constructed. The CXCR4 siRNA was driven by a U6 promoter whereas the CCR5 siRNA was driven by an H1 promoter. A CMV promoter driven EGFP reporter gene is also incorporated in the bispecific construct. High efficiency transduction into coreceptor expressing Magi and Ghost cell lines with a concomitant down regulation of respective coreceptors was achieved with lentiviral vectors. When the siRNA expressing transduced cells were challenged with X4 and R5 tropic HIV-1, they demonstrated marked viral resistance. HIV-1 resistance was also observed in bispecific lentiviral vector transduced primary PBMCs.

**Conclusions:**

Both CXCR4 and CCR5 coreceptors could be simultaneously targeted for down regulation by a single combinatorial lentiviral vector incorporating respective anti-coreceptor siRNAs. Stable down regulation of both the coreceptors protects cells against infection by both X4 and R5 tropic HIV-1. Stable down regulation of cellular molecules that aid in HIV-1 infection will be an effective strategy for long range HIV gene therapy.

## Background

HIV/AIDS continues to be a major public health problem worldwide with millions of people currently infected and new infections being on the rise. As no effective vaccines are currently available for prevention, new and innovative therapies need to be developed. Although combinatorial therapies such as HAART have proven to be effective in prolonging life, they do not afford a complete cure. Other constraints with HAART therapy are the development of drug resistant viral mutants and toxicity after prolonged therapy. Intracellular immunization by gene therapy strategies offers a promising alternative approach for controlling and managing HIV disease. A number of previous approaches that involved the use of transdominant proteins [1-3], decoys [3-7], and ribozymes [5,8-12] had shown initial promise but fell short of practical utility in providing adequate protection. With the discovery that the RNA interference phenomenon operates in mammalian cells and is highly effective in selective gene silencing, new potent small interfering RNA (siRNA) molecules have become available to add to the anti-HIV arsenal [13].

RNAi is a highly potent mechanism of post-transcriptional gene silencing. Mediated by sequence specific siRNAs, it can effectively down regulate expression of either viral or cellular RNA target molecules by selective degradation of mRNAs [13-16]. Mechanism of destruction involves an endonuclease present in the RISC complex which is guided by the antisense component of the siRNA for target recognition. A number of reports have shown that delivery of siRNAs by transfection of presynthesized or plasmids encoding siRNAs into cultured cells can effectively inhibit HIV-1 infections [17-26]. Antiviral effects of these delivery methods are only transient due to eventual degradation and dilution of siRNAs during cell division. For HIV gene therapy strategies to succeed in long range, it is necessary that siRNA coding transgenes be maintained and expressed long term in a virus susceptible target cell. In this regard, lentiviral vectors have proven to be highly effective in high efficiency gene transduction and sustained gene expression.

A number of previous approaches using either synthetic siRNAs or plasmid expressed constructs have successfully targeted viral transcripts and achieved effective viral inhibition. Of these, some anti-HIV-1 siRNAs, such as siRNAs against tat, tat-rev had been introduced into lentiviral vectors and their efficacy was demonstrated both in cell lines and primary T cells and macrophages [27,28]. Promising data was also obtained in experiments showing that anti-rev siRNAs against HIV-1 were functional in conferring viral resistance in differentiated T cells and macrophages derived from lentiviral transduced CD34+ hematopoietic progenitor cells [29].

In addition to targeting viral transcripts, many studies including ours also investigated the efficacy of siRNAs in down regulating host cell molecules necessary for HIV-1 infection [18,21,23,24,30,31]. An advantage in targeting cellular molecules is that efficacy will be more broad spectrum against all the clades of the virus and the frequency of escape mutants will be lower. Down regulation of the primary cell surface receptor CD4 and consequent inhibition of HIV-1 infection was shown using synthetic siRNAs. However, since CD4 is an essential cell surface molecule for immunological function, it is not a practical target for HIV gene therapy. Chemokine receptors CCR5 and CXCR4 play critical roles as coreceptors for viral entry during infection with macrophage tropic R5 and T cell tropic X4 HIV-1 viral strains respectively [32,33]. Thus they are suitable targets for siRNA mediated down regulation. Since both R5 and X4 viral strains are involved in disease pathogenesis, it is important to consider blocking of both respective coreceptors when developing effective therapeutics. In a segment of the human population, a naturally occurring 32-bp deletion in the CCR5 gene results in the loss of this coreceptor thus conferring significant resistance to HIV infection [34-36]. Homozygous or heterozygous individuals for this mutation remain physiologically normal. With regard to the CXCR4 coreceptor, it was found to be dispensable for T cell development and maturation in murine studies [37]. These findings suggest that CCR5 and CXCR4 are promising targets for HIV therapies.

Based on this rationale, recent work with synthetic siRNAs demonstrated that down regulating either CXCR4 or CCR5 will protect cells from X4 or R5 HIV-1 strains respectively at the level of viral entry [18,21,23,24]. Although stable expression of an anti-CCR5 siRNA was achieved using a lentiviral vector in one study, down regulating CCR5 alone in the face of an HIV-1 infection is insufficient [31]. Therefore, we recently experimented with synthetic bispecific combinatorial constructs targeted to both CXCR4 and CCR5 and have shown their efficacy in cultured cells [24]. To make further progress, our present studies are directed towards constructing a single bispecific lentiviral vector expressing both CXCR4 and CCR5 siRNAs. Using this combinatorial construct, here we show high efficiency transduction, simultaneous down regulation of both coreceptors resulting in HIV-1 resistance.

## Results and Discussion

### Coreceptor down regulation by a bispecific lentiviral vector

**Figure 1:**
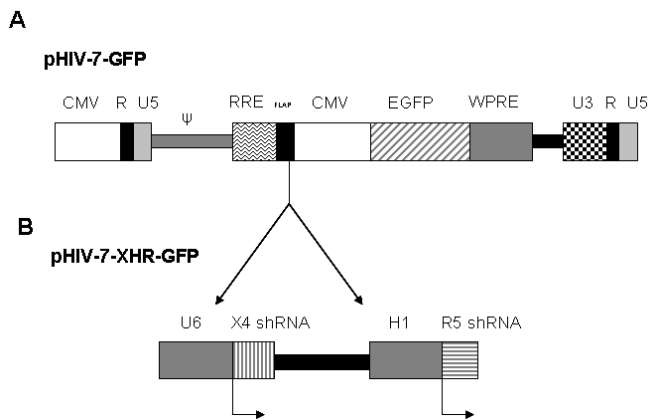
Bispecific lentiviral vector (XHR) encoding anti-CXCR4 and CCR5 siRNAs. A) Control transfer vector pHIV-7-GFP encoding a CMV promoter driven EGFP reporter gene. B) To derive the bispecific vector pHIV-XHR-GFP, a U6 promoter driven short hairpin CXCR4 siRNA cassette was cloned into the *BamH*I site upstream to the CMV-EGFP cassette. The H1-CCR5 siRNA cassette was inserted into an *Mlu*I site downstream to the U6-CXCR4 siRNA cassette.

Our major goal in these studies is to introduce both CXCR4 and CCR5 siRNAs into a single lentiviral construct to achieve their stable expression in transduced cells. Lentiviral vectors offer advantages over conventional retroviral vector systems since they can transduce dividing as well as nondividing cells and are less prone to transgene silencing [44-47]. The transfer vector HIV-7-GFP-XHR (referred to as XHR) contained a short hairpin type anti-CXCR4 siRNA driven by a Pol-III U6 promoter followed by a short hairpin anti-CCR5 siRNA driven by a different Pol-III promoter, H1. Downstream, the reporter gene, EGFP is driven by a CMV promoter. The control GFP-alone vector, HIV-7-GFP, contained only the reporter gene EGFP (Fig 1).

**Figure 2:**
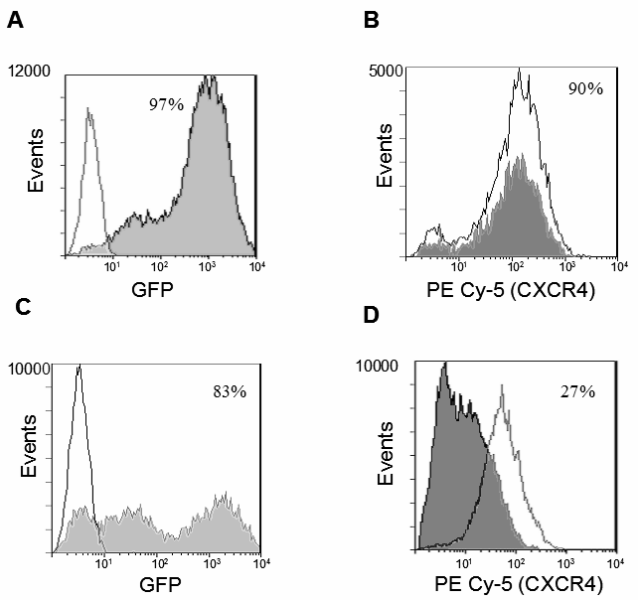
Cell surface down regulation of CXCR4 in XHR transduced Magi-CXCR4 cells. Magi-CXCR4 cells that constitutively express CXCR4 were transduced with control GFP or XHR vectors. Cells were stained with PECy5-conjugated antibodies to CXCR4 and analyzed by FACS 72 hours post-transduction. Levels of CXCR4 in non-transduced cells are superimposed (unshaded areas). Transduction efficiency was determined by FACS for EGFP expression. Levels of EGFP in control GFP-alone vector (A) and XHR vector (C) transduced cells. Levels of CXCR4 expression in GFP-alone (B) and XHR (D) vector transduced cells. Percent positive cells are indicated.

**Figure 3:**
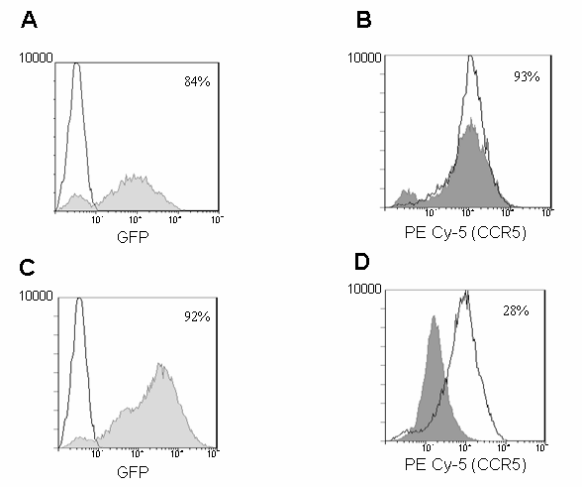
Cell surface down regulation of CCR5 in XHR transduced Ghost-R5 cells. Ghost-R5 cells that constitutively express CCR5 were transduced with GFP-alone or XHR vectors. Cells were stained with PECy5-conjugated antibodies to CCR5 and analyzed by FACS 72 hours post-transduction. Levels of CCR5 in non-transduced cells are superimposed (unshaded areas). Transduction efficiency was measured by FACS for EGFP expression. Levels of EGFP in control GFP-alone vector (A) and XHR vector (C) transduced cells. Levels of CCR5 expression in GFP-alone (B) and XHR (D) vector transduced cells. Percent positive cells are indicated.

Magi-CXCR4 cells constitutively expressing CXCR4 on the cell surface when transduced with the control vector or XHR vector had shown 97% and 83% EGFP expression respectively as measured by FACS analysis indicating high efficiency of transduction (Fig 2A and 2C). To determine if CXCR4 was down regulated by the respective siRNA in the XHR construct, the transduced cells were analyzed for CXCR4 surface expression. The surface levels of CXCR4 were reduced significantly in XHR transduced cells (73% lower) compared to the cells transduced with control vector (Fig 2B and 2D) indicating the efficacy of the CXCR4 siRNA on its target. Similarly, to determine the activity of the anti-CCR5 siRNA in the XHR vector, transduced Ghost R5 cells that constitutively express CCR5 were evaluated. As seen in Fig 3A and 3C, high levels of transduction (84% and 83%) were seen in Ghost-R5 cells with either the control vector or XHR vector, respectively. When the transduced cells were analyzed for CCR5 expression, a dramatic decrease in CCR5 expression was seen in XHR cells (72%) compared to control vector transduced cells (Fig 3B and 3D). These results had shown that the bispecific lentiviral vector XHR efficiently down regulates both CXCR4 and CCR5 targets in respective cells.

### Expression of siRNAs and down regulation of CXCR4 and CCR5 transcripts

**Figure 4:**
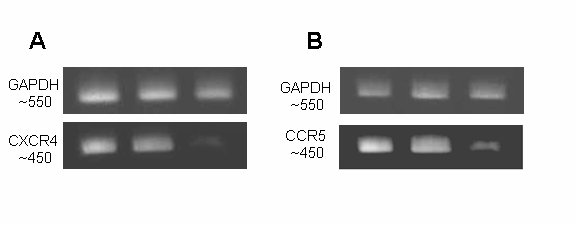
RT-PCR detection of CXCR4 and CCR5 mRNA down regulation. Total RNA was extracted from vector transduced cells and one-step RT-PCR was performed. PCR products of 450 bp were amplified to detect the coreceptor transcripts. A) Levels of CXCR4 mRNA in non-transduced (lane 1), GFP-alone (lane 2), and XHR (lane 3) vector transduced Magi-X4 cells. B) CCR5 transcript levels in non-transduced (lane 1), GFP-alone (lane 2), and XHR vector transduced Ghost-R5 cells. GAPDH transcript levels were used as internal controls (PCR product size ~550 bp).

**Figure 5:**
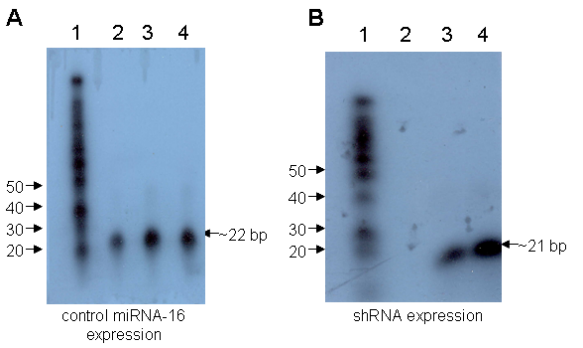
Northern analysis to detect siRNA expression in transduced cells. Small RNAs (<200 nt) were extracted from transduced cells and probed with specific primers to detect the expression of siRNAs as described in materials and methods. A) Northern blot to detect the presence of miRNA-16 (~22 bp) as an internal control in GFP-alone vector transduced (lane 2) and XHR transduced Magi-X4 (lane 3) and Ghost-R5 (lane 4) cells. B) siRNA (~21 bp) detection in GFP-alone vector transduced (lane 2) and XHR transduced Magi-X4 (lane 3) and Ghost-R5 (lane 4) cells. Decade markers (lanes A1 and B1).

To confirm that the down regulation of both CXCR4 and CCR5 coreceptors as seen by FACS analysis is due to reduced levels of the corresponding mRNAs, vector transduced cells were analyzed by RT-PCR. As an internal control, GAPDH mRNA was also analyzed. XHR vector transduced cells showed considerable reduction in transcript levels for both CXCR4 and CCR5 as compared to non-transduced and control GFP vector transduced cells. The levels of GAPDH control mRNA remained unchanged in all samples (Fig 4). To validate the expression of individual siRNAs in transduced Magi-CXCR4 and Ghost R5 cells, cellular RNA was analyzed by northern analysis for their presence. As internal controls, the presence of constitutively expressed miRNA-16 RNAs were also analyzed in parallel. As expected, comparable levels of miRNA-16 RNAs (22 bp in length)were detected in GFP control vector transduced as well as in XHR vector transduced cells (Fig 5A). RNAs corresponding to CXCR4 and CCR5 shRNAs (representing the 21nt antisense strand of each shRNA) were seen in XHR transduced but not in GFP control vector transduced cells (Fig 5B).

### Bispecific siRNA vector does not induce interferon

**Figure 6:**
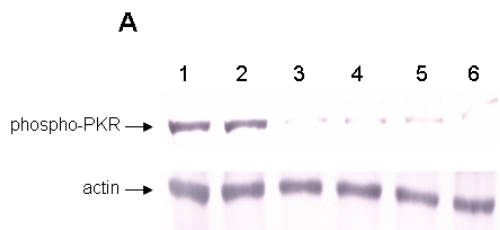
Lack of interferon induction in siRNA transduced cells. To detect interferon induction in siRNA vector transduced cells, western blot analysis was performed to detect elevated levels of phophorylated PKR. Poly I:C was used to induce interferon as a positive control. Transduced cell extracts were run on 10% SDS-PAGE gels, transferred, and probed with an anti-phospho-PKR antibody. Positive control poly I:C transfected (lanes 1 and 2), non-transduced (lane 3), GFP-alone vector (lane 4), and XHR transduced (lane 5) Magi-X4 cells and XHR transduced Ghost-R5 cells (lane 6). An anti-actin antibody was used as an internal control.

Double stranded RNA molecules longer than ~30 bp are known to induce the interferon pathway in response to viral infections. As siRNAs are generally comprised of 19–24 bp in length, they are not expected to activate such a response that mediates a non-specific down regulation of cellular or viral mRNAs. However, recent data had shown that in some circumstances, certain siRNAs might induce variable levels of interferon activation [48-50]. To rule out such a possibility with the present siRNAs, we looked for upregulation of phosphorylated-PKR by western blot analysis. PKR is a protein kinase that becomes activated through phosphorylation in the presence of dsRNA and is involved during the interferon response. Our results have shown that the levels of phosphorylated PKR remain unchanged in XHR transduced cells similar to mock and GFP vector transduced cells. In contrast, elevated levels of phosphorylated PKR could be seen in poly I:C transfected cells used as positive controls (Fig 6). These data exclude the possibility of non-specific interferon activation by the combinatorial lentiviral construct.

### Resistance of siRNA transduced cells to HIV-1 infection

**Figure 7:**
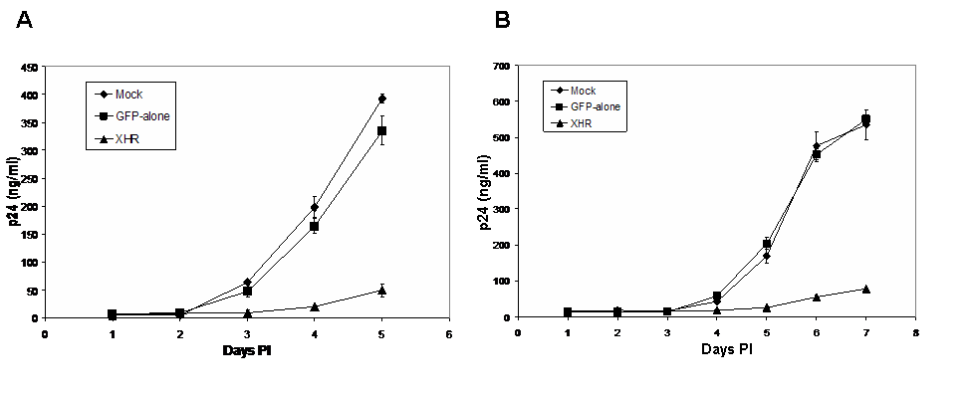
HIV-1 challenge of XHR transduced Magi-X4 and Ghost-R5 cells. Vector transduced cells were challenged with either X4 tropic or R5 tropic viruses at an m.o.i of 0.01. Culture supernatants were collected at different days post challenge and p24 antigen was assayed by ELISA. A) Transduced Magi-X4 cells challenged with X4 tropic HIV-1 NL4-3. B) Transduced Ghost-R5 cells challenged with R5 tropic HIV-1 BaL-1. Data presented is from triplicate experiments.

**Figure 8:**
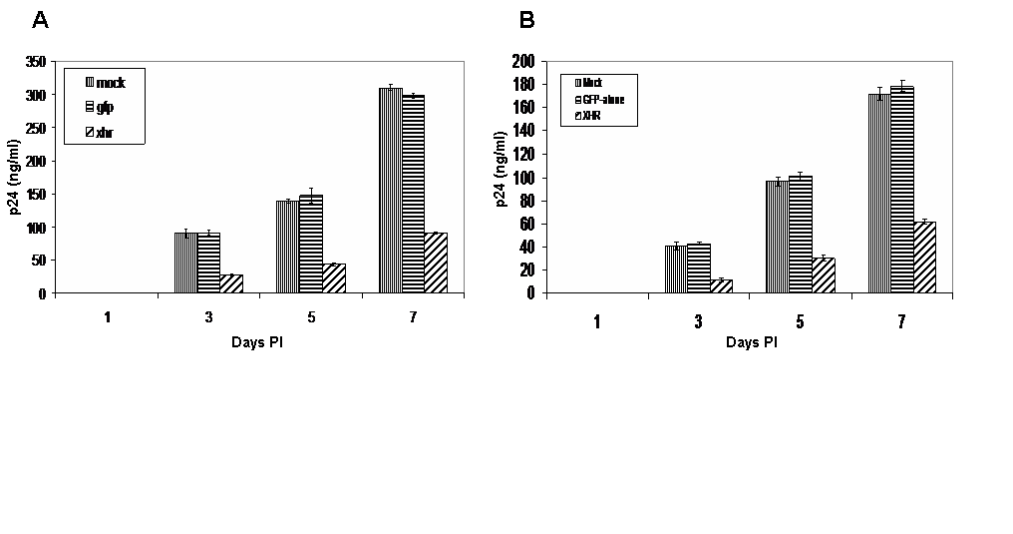
HIV-1 challenge of XHR transduced PBMCs. Vector transduced PBMCs were challenged with either X4 tropic or R5 tropic viruses. Culture supernatants were collected at different days post challenge and p24 antigen was assayed by ELISA. Transduced PBMCs challenged with either HIV-1 NL4-3 (A) or BaL-1 (B). Data presented is from triplicate experiments.

To determine if down regulation of the essential coreceptors, CXCR4 and CCR5, translated to virus resistance, transduced Magi-CXCR4 and Ghost R5 cells were challenged with X4 (NL4-3) and R5 (BaL1)-tropic strains of HIV-1 respectively. Viral p24 antigen levels at different days post-challenge were determined by ELISA to quantify levels of HIV-1 resistance. Over a 10-fold reduction in viral antigen levels was seen with both XHR transduced Magi-CXCR4 and Ghost-R5 cells as compared to non-transduced and GFP-alone vector transduced cells (Fig 7). There was a slight increase in viral production in XHR transduced cells on days 5 to 7. This could be due to nontransduced and/or low siRNA expressing cells producing the virus. We next wanted to determine if the XHR vector expressing CXCR4 and CCR5 siRNAs is effective in physiologically relevant cells for gene therapy. Accordingly, PBMCs transduced with vectors were challenged in the same manner as above. A 3-fold level of inhibition was seen on days 3, 5, and 7 (Fig 8). These results established that the XHR vector is also effective in primary cells in inhibiting HIV-1. Although clearly significant, the levels of virus inhibition were not as dramatic as seen with Magi and Ghost cell lines. The observed levels of viral inhibition in primary PBMC are similar to those observed in a recent report [31]. Lower levels of protection in PBMCs were likely due to the lower levels of transduction. Future studies that are aimed at increasing transduction efficiencies into primary lymphocytes and macrophages are likely to overcome this hurdle.

In summary, our studies have shown for the first time that a single lentiviral vector could be used to stably deliver two different siRNAs targeted to two different cell surface co-receptor molecules and achieve protection against both X4 and R5 tropic HIV-1 viral strains. The short hairpin design permitted use of a single promoter to transcribe both the sense and anti-sense strands of each of the siRNAs. No promoter interference was observed between the U6 promoter driving the transcription of CXCR4 siRNA and the H1 promoter driving the CCR5 siRNA since comparable amounts of both the siRNAs could be seen in transduced cells. Furthermore, possible interferon induction by the combinatorial construct was also ruled out.

A major advantage in using a combinatorial lentiviral construct targeted to both the coreceptors is that infection with either of the viral strains could be prevented at the entry step thus eliminating the possibility of proviral integration and viral latency. Given the success with the current bispecific construct, other novel constructs could be designed and experimented with that incorporate siRNAs targeted to both the cellular as well as viral targets. Based on the design employed here, it is possible to introduce more than two siRNAs in a single construct in the future. However caution should be exercised while incorporating multiple siRNAs in a single construct because the possibility exists that over expression of foreign siRNAs in a cell may have undesirable effects such as saturating the endogenous RISC complex and consequent toxicity. Such a possibility needs to be tested in long range experiments *in vivo*. We previously have introduced a monospecific siRNA targeted to HIV-1 *rev *into CD34 hematopoietic progenitor cells via lentiviral vectors and derived transgenic macrophages *in vitro *and T cells *in vivo *[29]. The transgenic cells were found to be apparently normal while markedly resistant to HIV-1 infection.

No deleterious effects are expected by the stable knock down of the CCR5 coreceptor *in vivo *since individuals harboring a 32 bp deletion in the corresponding gene are physiologically normal [34,35]. Although CXCR4 down regulation in circulating mature T cells in the periphery may not have any insurmountable ill effects, this may have possible drawbacks in a stem cell setting due to its role in cell homing into bone marrow [51,52]. Additionally, recent gene expression profiling studies indicated some off-target effects by siRNAs [53]. Therefore, the present combinatorial construct targeted to both CXCR4 and CCR5 coreceptor molecules need to be thoroughly tested in an *in vivo *system such as the SCID-hu mouse model to evaluate its efficacy and possible toxicity in differentiated cells before it can be used for gene therapy in human subjects. Such experiments are currently underway.

## Conclusions

For HIV/AIDS gene therapy strategies to succeed, novel molecules need to be harnessed. In this regard, siRNAs offer great potential. Exploitation of these promising candidates to down regulate essential cellular coreceptors via the use of lentiviral vectors facilitates long term derivation of resistant T cells and macrophages which are the main targets for the virus. Our results showed for the first time that expression of both CXCR4 and CCR5 siRNAs in combination is possible by the use of lentiviral vectors. Coreceptor specific siRNAs stably transduced with the bispecific lentiviral vector showed marked resistance against both T cell tropic and monocyte tropic HIV-1 infection in cell lines and primary PBMCs. The newly developed bispecific vector shows promise for potential *in vivo *application.

## Materials and Methods

### Plasmid and lentiviral vector construction

Previously characterized siRNAs against CXCR4 and CCR5 were used in generating the bispecific lentiviral vector [23,24,30]. A third generation lentiviral vector backbone was employed to derive the bispecific constructs. The two *cis*-acting elements, namely, the central DNA flap consisting of cPPT and CTS (to facilitate the nuclear import of the viral preintegration complex) and the WPRE (to promote nuclear export of transcripts and/or increase the efficiency of polyadenylation of transcripts), are engineered to enhance the performance of the vector [38,39]. An siRNA expression cassette targeting CXCR4 under the control of the Pol-III U6 promoter was PCR amplified from the plasmid pTZ-U6+1 as described by Castanotto *et al *[40]. This cassette was cloned into pHIV-7-GFP transfer vector in the *BamH*I site immediately upstream of the CMV-EGFP gene. This cassette contained a *Mlu*I restriction site downstream from the CXCR4 siRNA sequence for subsequent cloning of the H1 promoter driven CCR5 siRNA cassette. The H1-CCR5 siRNA expression cassette was also generated as described above using the plasmid pSUPER (Oligoengine, Seattle, WA). Sequencing and confirmation of candidate clones was performed by Laragen Inc. (Los Angeles, CA). The transfer vector containing the inserts U6-X4 siRNA and H1-CCR5 siRNA is termed pHIV-XHR-GFP.

### Cell culture and vector production

293T cells and PBMCs were maintained in DMEM media supplemented with 10% FBS. Magi-CXCR4 cells obtained from the AIDS Reference and Reagent Program were maintained in media as previously described [41,42]. Ghost-R5 cells obtained from the AIDS Reference and Reagent Program were maintained in media as previously described [43]. To generate lentiviral vectors, fifteen micrograms of transfer vector with either GFP-alone or XHR were transfected along with 15 ug pCHGP-2, 5 ug pCMV-Rev, and 5 ug pCMV-VSVG into 293T cells at 60% confluency in 100 mm culture dishes using a calcium phosphate transfection kit (Sigma-Aldrich, St. Louis, MO). Six hours after transfection, fresh medium was exchanged. Cell culture supernatants containing the vector were collected at 24, 36, 48, and 60 hours post transfection and pooled. Vector supernatants were concentrated by ultracentrifugation and later titrated on 293T cells using FACS analysis for GFP expression.

### Lentiviral vector transduction and FACS analysis

Magi-CXCR4 and Ghost-CCR5 cells were seeded in 6-well plates 24 hours prior to transduction, 5 × 10^5 ^cells per well. Cells were transduced with lentiviral vectors at an m.o.i. of 10 in the presence of 4 ug/ml polybrene for 2 hours. For transduction of PBMCs, cells were first isolated from whole blood by Histopaque^®^-1077 (Sigma-Aldrich), and then cultured in CD3 and CD28 antibody coated plates. Three days after stimulation, PBMCs were transduced at an m.o.i of 20 in the presence of 4 ug/ml polybrene. PBMC transduction was repeated the following day. Seventy-two hours post transduction with siRNA containing lentiviral vectors, FACS analysis was performed to determine the levels of cell surface expression of CXCR4 and CCR5. Non-transduced and transduced cells were stained with appropriate antibodies conjugated with PE-Cy 5 (Pharmingen, San Diego, CA) namely, anti-CXCR4 for Magi-CXCR4 cells and anti-CCR5 for Ghost-CCR5 cells. Transduction efficiency was determined by assaying for EGFP expression. FACS analysis was performed on the Beckman Coulter Epics XL using ADC software for analysis.

### Northern analysis for shRNA expression

Total RNA was extracted from non-transduced and transduced Magi-CXCR4 and Ghost-CCR5 cells using the RNA-STAT-60 reagent (Tel-Test, Friendswood, TX). Small RNAs, <200 nt, were separated and concentrated using the *mir*Vana™ miRNA Isolation Kit (Ambion, Austin, TX). Twenty micrograms of small RNAs were hybridized overnight at 37°C using the *mir*Vana™ miRNA Detection Kit (Ambion) with γ-^32^P labeled probes made using the *mir*Vana™ Probe & Marker Kit (Ambion). Probes were complementary to the antisense strands of CXCR4 and CCR5 siRNAs. Hybridization reactions were processed according to the manufacturer's protocol and run on 15% polyacrylamide TBE-Urea gels. Gels were then exposed to X-ray film. A probe complementary to miRNA-16 supplied with the miRNA detection kit was used as an internal control.

### Western Blot analysis of phosphorylated PKR

Cell lysates of non-transduced and transduced cells were run on 10%-polyacrylamide-SDS TBE gels. Proteins were immunoblotted onto Immobilon™-P membranes (Millipore, Bedford, MA) and incubated with antibody specific for phosphorylated-PKR (Sigma-Aldrich), while anti-actin antibody (Sigma-Aldrich) was used to detect cellular actin as an internal control. A secondary antibody, goat anti-rabbit IgG conjugated with alkaline phophatase (Promega, Madison, WI), was then added. An alkaline phophatase substrate reagent, Western Blue (Promega), was used to visualize the bands.

### RT-PCR

Total RNA was extracted from non-transduced and transduced cells. Primers specific for CXCR4 (forward: 5'-ggaggggatcagtatatacacttc and reverse: 5'-cgccaacatagaccaccttttc) and CCR5 (forward: 5'-caaaaagaaggtcttcattacacc and reverse: 5'-cttgctcgctcgggagcctc) (IDT, Coralsville, IA) were used to determine transcript levels while GAPDH (forward: 5'-ctgagaacgggaagcttgtcatcaa and reverse: 5'-gcctgcttcaccaccttcttgatg) primers were used as an internal control. One-step RT-PCR reactions were performed using the Superscript™ III One-Step RT-PCR kit (Invitrogen, Carlsbad, CA). Reactions were run on 1% agarose gels and appropriate bands were visualized with UV light.

### HIV-1 Challenge

To determine if down-regulation of CXCR4 and CCR5 transcript levels and cell surface expression inhibited HIV-1 infection, non-transduced and transduced cells were challenged with NL4-3 (X4-tropic) and BaL-1 (R5-tropic) strains of HIV-1, at an m.o.i of 0.01, as previously described [24]. Viral supernatants were collected daily from infected Magi-CXCR4 and Ghost-CCR5 cells for p24 assay. ELISA was used to determine p24 values employing a Coulter-p24 kit (Beckman Coulter, Fullerton, CA). For PBMC challenge experiments, non-transduced and transduced cells were infected with NL4-3 and Bal-1 strains and cell culture supernatants were collected on days 1, 3, 5, and 7 post-infection to measure p24 levels.

## Competing interests

The author(s) declare that they have no competing interests.

## Author's contributions

JA carried out all of the experiments. RA was responsible for the overall experimental design and implementation of the project.
